# Exposure to GSM RF Fields Does Not Affect Calcium Homeostasis in Human Endothelial Cells, Rat Pheocromocytoma Cells or Rat Hippocampal Neurons

**DOI:** 10.1371/journal.pone.0011828

**Published:** 2010-07-27

**Authors:** Rodney P. O'Connor, Steve D. Madison, Philippe Leveque, H. Llewelyn Roderick, Martin D. Bootman

**Affiliations:** 1 Laboratory of Molecular Signalling, The Babraham Institute, Cambridge, United Kingdom; 2 XLIM, University Limoges, Limoges, France; 3 Department of Pharmacology, University of Cambridge, Cambridge, United Kingdom; Karolinska Institutet, Sweden

## Abstract

In the course of modern daily life, individuals are exposed to numerous sources of electromagnetic radiation that are not present in the natural environment. The strength of the electromagnetic fields from sources such as hairdryers, computer display units and other electrical devices is modest. However, in many home and office environments, individuals can experience perpetual exposure to an “electromagnetic smog”, with occasional peaks of relatively high electromagnetic field intensity. This has led to concerns that such radiation can affect health. In particular, emissions from mobile phones or mobile phone masts have been invoked as a potential source of pathological electromagnetic radiation. Previous reports have suggested that cellular calcium (Ca^2+^) homeostasis is affected by the types of radiofrequency fields emitted by mobile phones. In the present study, we used a high-throughput imaging platform to monitor putative changes in cellular Ca^2+^ during exposure of cells to 900 MHz GSM fields of differing power (specific absorption rate 0.012–2 W/Kg), thus mimicking the type of radiation emitted by current mobile phone handsets. Data from cells experiencing the 900 Mhz GSM fields were compared with data obtained from paired experiments using continuous wave fields or no field. We employed three cell types (human endothelial cells, PC-12 neuroblastoma and primary hippocampal neurons) that have previously been suggested to be sensitive to radiofrequency fields. Experiments were designed to examine putative effects of radiofrequency fields on resting Ca^2+^, in addition to Ca^2+^ signals evoked by an InsP_3_-generating agonist. Furthermore, we examined putative effects of radiofrequency field exposure on Ca^2+^ store emptying and store-operated Ca^2+^ entry following application of the Ca^2+^ATPase inhibitor thapsigargin. Multiple parameters (e.g., peak amplitude, integrated Ca^2+^ signal, recovery rates) were analysed to explore potential impact of radiofrequency field exposure on Ca^2+^ signals. Our data indicate that 900 MHz GSM fields do not affect either basal Ca^2+^ homeostasis or provoked Ca^2+^ signals. Even at the highest field strengths applied, which exceed typical phone exposure levels, we did not observe any changes in cellular Ca^2+^ signals. We conclude that under the conditions employed in our experiments, and using a highly-sensitive assay, we could not detect any consequence of RF exposure.

## Introduction

All mammalian cells experience regular pulses of calcium (Ca^2+^) in response to cues such as electrical depolarisation, hormones and mechanical deformation [1], [2]. Ca^2+^ modulates a wide range of cellular activities, including gene transcription, metabolism and programmed cell death [3]. The Ca^2+^ signals that control these processes can arise via release of the ion from intracellular stores, or activation of specific channels that gate flux of Ca^2+^ from the extracellular space into the cellular volume [4]. As Ca^2+^ signals are critical in transducing the cellular effects of physiological stimuli, aberrant Ca^2+^ signals can lead to pathological changes in cell behaviour [5], [6].

In recent years, the widespread use of mobile phones has been accompanied by concern over their possible effects on health (for a review see Independent Expert Group on Mobile Phones, http://www.iegmp.org.uk/report/index.htm). Some of this concern was fostered by the results of experiments performed 30–40 years ago, which examined the biological effects of radio frequency (RF) waves on the brain, and in particular on the influence of modulated RF fields on Ca^2+^ fluxes. Adey and colleagues reported that RF electromagnetic fields influenced Ca^2+^ within tissues following experiments measuring ^45^Ca^2+^ fluxes in excised chick brain [7]. They exposed whole chick cerebral hemisphere samples to RF fields using a parallel plate apparatus, and found a statistically significant increase in ^45^Ca^2+^ efflux in samples exposed to specific RF signals.

Consistent with these findings, an independent group from the US Environmental Protection Agency performed a series of experiments that reproduced and extended the previously observed effects of RF exposure on Ca^2+^ efflux in chick brain samples [8]. A statistically significant increase in Ca^2+^ efflux was observed in samples exposed to RF fields. The increase in Ca^2+^ efflux was found to be dependent on the power density of the RF exposure, occurring at 0.75 mW/cm^2^ and not at the higher or lower powers used. The importance of specific power density was also reported by Adey and colleagues [9]. Blackman and colleagues confirmed and extended their findings in later studies, and observed that several combinations of frequencies and intensities enhanced Ca^2+^ efflux from chick brain hemispheres [10], [11].

Subsequent studies have failed to replicate the results of Adey and co-workers. In particular, experiments using rat brain extracts preloaded with ^45^Ca^2+^ did not reveal a change in Ca^2+^ efflux over a range of power densities [12]. This study is often cited as a failed replication of the original findings from Adey and colleagues, but, the radiation exposure parameters used a higher RF carrier frequency and digital modulation. However, these parameters are more relevant to current mobile phone signals than the lower frequency, amplitude-modulated RF exposures employed by Adey and others. Similarly, Merritt and colleagues failed to find an effect of RF fields on ^45^Ca^2+^ efflux following *in vivo* loading of the ion, using both whole animal and brain tissue exposure methods [13]. Other independent groups have also been unsuccessful in reproducing the original findings of Adey and Blackman [14].

In addition to studies using excised brain tissues, putative effects of RF fields have been examined using cultured neurons where experimental conditions can be rigorously controlled. For example, exposure of ^45^Ca^2+^-loaded neuroblastoma cells to amplitude-modulated RF fields, at particular power densities (e.g. 0.05 W/kg) and modulation frequencies (e.g. 16 Hz), was found to have a significant effect on Ca^2+^ ion efflux [15], [16]. The authors concluded from these studies that neuroblastoma cell lines exhibit the same sensitivity to RF exposure as the forebrain preparations previously used by Adey and Blackman.

The development of fluorescent indicators capable of reporting intracellular Ca^2+^ ion concentration [17] allowed a new series of RF exposure experiments to be performed using fluorescence microspectroscopy methods. By the time these fluorophores were implemented in RF research, some of the focus had moved onto effects of pulsed RF radiation associated with mobile telephones, and in particular the signals produced by handsets using the GSM (Global System for Mobile Communications) modulation standard.

Meyer and colleagues examined Ca^2+^ changes in cardiac myocytes in the presence of GSM pulsed RF fields, and reported a small effect when the cells were exposed to 900 MHz fields that were modulated at 50Hz [18]. However, a further study by the same group failed to observe any significant effects of GSM pulsed RF fields on cardiac Ca^2+^ currents [18]. In this case, exposure was performed at a wider range of power densities using either a transverse electromagnetic (TEM) cell exposure system or a rectangular waveguide. More recently, fluorescent indicators have been used to study the influence of pulsed RF fields on intracellular Ca^2+^ in cultured neurons [19]. Tattersall and colleagues performed a well-controlled series of electrophysiological experiments examining the influence of pulsed RF fields on neuronal excitability [20]. They observed that acute exposure of hippocampal slices to continuous wave (CW) 700 MHz fields led to changes in evoked and spontaneous electrical activity at power densities as low as 1.6 mW/kg. In follow up experiments, cellular Ca^2+^ was also monitored in cultured rat cerebellar granule cells and cardiac myocytes. The biological effects of both CW and TETRA (Terrestrial Trunked Radio) pulsed RF fields were studied at a carrier frequency of 380 MHz and a wide range of power densities (5–400 mW/kg) [19]. No significant changes in intracellular Ca^2+^ were observed in cells exposed to either the CW or TETRA pulsed RF conditions.

The studies described above illustrate a discrepant and controversial literature concerning the impact of RF fields on Ca^2+^ homeostasis in mammalian cells. Given that Ca^2+^ is a critical cellular messenger, it is vitally important to establish whether such an interaction is plausible. We therefore applied a novel microscope-based high throughput screening system that allowed the automatic analysis of mammalian cells under different exposure regimes. In addition, we used fluorescent indicators that provide high sensitivity and good signal to noise reporting of cellular calcium concentration in within living cells.

## Materials and Methods

### Ethics statement

All experiments conformed to guidelines laid out by the UK Home Office; animals were killed using authorised Schedule 1 procedures according to Home Office guidelines. Furthermore, all procedures were approved by Babraham Institute Animal Welfare and Ethics Committee.

### Imaging-based screening system

**Figure 1:**
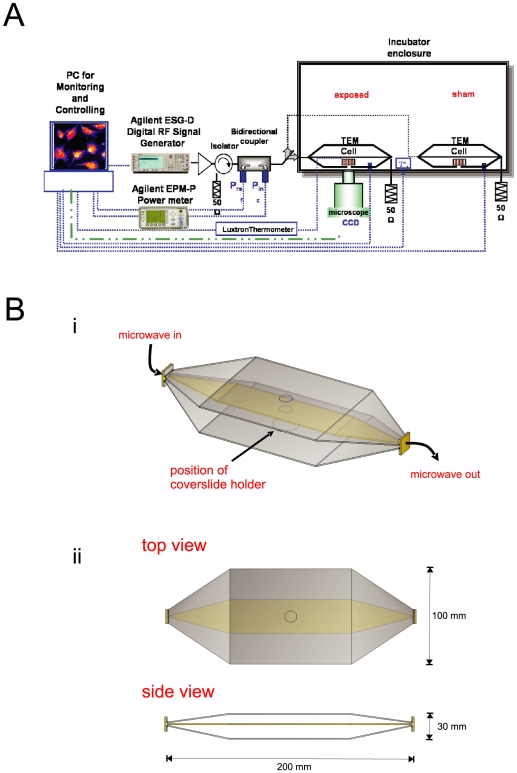
An Imaging-based System for Screening the Cellular Effect of RF Fields. **A**; The diagram depicts the screening system configured for the dual TEM cell mode of operation. This configuration permitted two imaging experiments to be carried out simultaneously, with the microscope collecting fluorescence images by alternating between each of the two independent TEM cells. A personal computer controlled the exposure conditions by setting the amplified power output of the Agilent ESG-D RF Signal Generator to one of the two TEM cells, through an in-line bidirectional coupler and an RF switch. An Agilent EPM-P Power meter measured the incident and reflected power at each TEM cell. Each TEM cell was terminated with a 50 Ohm load. The exposure and imaging were carried out in an environmental enclosure, where temperature, CO_2_ and humidity were controlled. **Bi**; The transverse electromagnetic cell exposure system used TEM cells with open sides. This design permitted the equilibration of temperature, humidity and gases with the environmental enclosure system during the incubation. RF power was delivered to the samples in the TEM cell by SMA connectors and then terminated in a 50 Ohm load. Coverslides were mounted in Teflon chambers and positioned at the centre of the TEM cell between the septum and the ground plate. An aperture in the ground plate allowed optical access for imaging from below, while apertures in the septum above and top plate permitted the addition of compounds, as required in some experiments. A retaining ring around the aperture permitted exact placement of the coverslide within the TEM cell. **Bii**; The dimensions of the TEM cell and apertures were optimized for coupling RF power into the TEM cell to achieve homogenous, far-field conditions at the sample.

A customized version of the Discovery-1 (Molecular Devices Corp., Downingtown, PA) fluorescence imaging screening system was used in all experiments. A schematic drawing of all the major components is presented in figure 1A. The imaging system was based around a motorized, inverted Nikon microscope. The stage of the imaging system was replaced with a customized motorized stage (Prior). Fluorescence illumination and collection was controlled with a series of motorized excitation, emission and dichroic filter wheels. Images were captured with a 12-bit CCD camera (Coolsnap HQ, Photometrics) and saved to a PC. The system was controlled by Metamorph using the Discovery-1 HTS module, which controlled the imaging and stage movement, logged experimental conditions and saved image stacks in a database. A custom-made environmental control chamber was constructed (Babraham Electronics and Mechanical Workshop) to enclose the imaging system and maintained temperature at 37±0.1°C by circulating air within the enclosure. The relative humidity of the air was maintained using a water tray, which was placed in the enclosure. The environmental chamber also provided a gas control system permitting the CO_2_ atmosphere of the imaging chamber to be monitored, controlled and matched to the buffers appropriate for the cell types under study.

### Design and Implementation of the RF exposure system

**Figure 2:**
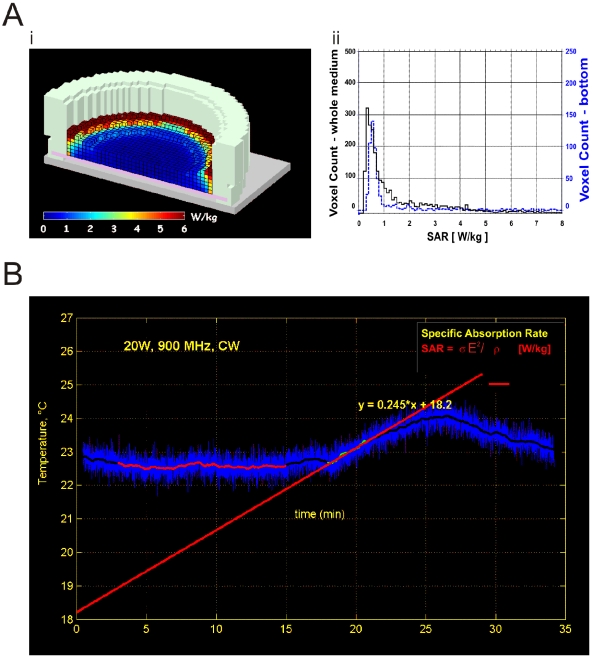
Computational and Experimental Estimates of the SAR Distribution in the Imaging Chamber. **Ai**; the imaging chamber consisted of a 22 mm borosilicate coverslide (with adherent cells) sandwiched between a bevelled Teflon cylinder and retaining ring. The imaging chamber held a volume of 0.5 ml of HBSS. The finite-difference time domain (FDTD) method was used to compute the SAR distribution in the imaging chamber. A pseudocolored image of the distribution shows the homogeneity of SAR across the media, with the exception of gradients at the meniscus. **Aii**; A histogram of the SAR distribution is shown for both the whole media (black solid line) and at the bottom of the media near the location of adherent cells (blue dotted line). **B**; The SAR in the media per Watt of input power was determined experimentally by measuring the temperature change in the sample with a fluoroptic temperature probe when exposed to an incident continuous power of 20 W. The SAR was derived from the change in temperature (ΔT°) produced over time (Δt), given the specific heat capacity (*C*) of the sample, following SAR = *C* ΔT°/Δt [W/kg]. The resulting SAR of the exposure configuration was estimated as 0.85 W/kg per W of incident power.

The RF exposure system was based around an open transverse electromagnetic (TEM) cell (Figure 1B). Similar TEM cells have been used in previous studies [21]. The final exposure system consisted of 2 identical TEM cells that were designed to hold a single coverslip each (22 mm diameter) in a Teflon ring sandwich chamber. An open design was chosen for the TEM cell design to achieve suitable control of temperature and ambient atmosphere during the imaging experiments. The bottom conductor of the TEM cell contained hole to allow optical access to the cells for imaging purposes. The TEM cell exposure setup was suitable for exposure frequencies from DC to 1 GHz and allowed generation of far-field conditions at the centre of the waveguide where the sample was positioned. The specific absorption rate (SAR) of the TEM cell containing the imaging chamber and buffered salt solution was estimated using both computational and experimental methods. The RF field simulation software was based on the finite-difference time-domain (FDTD) method [22]. A non-uniform mesh was used to compute the dish and TEM Cell volume. The smallest voxel was 0.2×0.2×0.1 mm. A perfectly-matched layer absorbing boundary condition was used around the structure [23]. Results of the FDTD simulation are shown on figure 2, where the histogram of the SAR distribution in the whole media and at the bottom of the imaging chamber are shown. The FDTD model provided an estimate of the SAR at 0.65 W/kg per 1 W incident power. Further details of the FDTD model and software can be obtained from Dr Philippe Leveque (XLIM, Limoges, France; email: philippe.leveque@unilim.fr). An experimental estimate of the SAR was also obtained by measuring the temperature change in the sample with a fluoroptic temperature probe (Model 501, Luxtron) when exposed to a continuous power of 20 W. The SAR was derived from the change in temperature (ΔT°) produced over time (Δt), given the specific heat capacity (*C*) of the sample and following the relation, SAR = *C* ΔT°/Δt [W/kg], as shown in figure 2B. The resulting SAR of the exposure configuration was estimated as 0.85 W/kg per W of incident power. A further estimate of the SAR was derived experimentally with an electric field probe by Dr. Benjamin Loader and colleagues at the National Physical Laboratory (Teddington, UK), who found an SAR of 0.66 W/kg, in close agreement with our findings.

The TEM cells were mounted side by side on the motorized stage of the Discovery-1 microscope. The arrangement of the TEM cells was chosen to facilitate placement of the mounted coverslips into the microscope light path. Acquisition software alternated the image capture between the two TEM cells during the course of an experiment. In this way, exposed and sham-exposed samples were subjected to identical physical conditions. An Agilent E4432B Signal Generator produced an industry standard GSM 900 MHz (1/8 time) signal with a 217 Hz recurrence frequency, as well as the 900 MHz continuous wave (CW) signal when GSM modulation was toggled off. The state of the RF switch was set and logged by software, which directed the signal into either the left-hand or right-hand TEM line. A vector network analyzer was used to measure the isolation between the two TEM cells and evaluate whether the RF field in the energized condition could influence the other TEM cell. The transmission coefficient between the two TEM cells was −30 dB (data not shown), indicating a negligible interaction.

The majority of experiments used 900 MHz GSM moduated RF fields. The SARs of handsets differ; however, mobile phones sold in the UK produce an average SAR of less than 2 W/kg to meet guidelines set by ICNIRP [24]. For this reason, a maximal SAR of 2 W/kg was chosen for experiments. Some experiments were also performed at SARs between 12 mW/kg and 2 W/kg to search for potential effects at lower powers. An Agilent E4417A EPM-P Series Power Meter was used for the continuous monitoring of the incident and reflected power supplied to the TEM cells.

### Cell culture

EA.hy926 human endothelial cells (kindly donated by Dr. Cora-Jean Edgell, University of North Carolina, Chapel Hill, North Carolina) were cultured in Dulbecco's modified Eagle's medium (DMEM; Invitrogen) supplemented with 10% foetal bovine serum (Invitrogen) and 2% L-Glutamine-Penicillin-Streptomycin solution (Sigma). In an attempt to reduce variability, a single batch of foetal bovine serum was used to supplement the culture media used during all experiments. Cells were seeded at an initial density of 1–2×10^5^ cells per ml in 25 cm^2^ culture flasks (Nunc). Cells were maintained under standard culture conditions (i.e. constant 37°C temperature, humidity of 95%, and 5% CO_2_) in a conventional culture incubator. Fresh media was introduced every 3–4 days and the cell line maintained by reseeding 1–2×10^5^ cells per ml into a fresh 25 cm^2^ flask during the exponential growth phase. At the same time, ∼2–4000 cells were seeded onto 22 mm glass coverslips (VWR International) and placed into individual 35 mm culture dishes (Nunc) containing 2 ml of fresh culture media. Cells were allowed to grow to 80% confluence.

PC12 cells were obtained from the American Type Culture Collection, and have been described previously [25], [26], [27]. The cells were cultured in DMEM (Invitrogen) supplemented with 10% foetal bovine serum (Gibco), 5% horse serum (Gibco) and 2% L-Glutamine-Penicillin-Streptomycin Solution (Sigma) and maintained under standard culture conditions. PC12 cells were differentiated with NGF and all experiments were conducted at ∼20% confluence. Fresh media was introduced every 3–4 days and cells seeded onto 22 mm glass coverslips. For differentiation, PC12 cells were incubated for 2 hours in serum-free DMEM, and NGF (50 ng/ml) was added to the cultures. The cells were allowed to incubate for a further 48 hours before imaging.

Primary rat hippocampal neurons (isolated from rats postpartum day 2–3) were prepared using established procedures and seeded onto 22 mm coverslips [25]. Cells were housed under standard culture conditions for 3 days in plating media consisting of Neurobasal Media (Invitrogen) supplemented with 2% B27 Supplement (Invitrogen), GSP cocktail (0.5 mM L-glutamine, 100 µM L-serine and 1 mM pyruvate) and 10% Foetal Bovine Serum (Invitrogen, not heat inactivated). Cells were then switched to a Basal Feeding Medium consisting of Neurobasal Media (Invitrogen) supplemented with 2% B27 Supplement (Invitrogen) and the GSP cocktail. Fresh media was introduced every 4 days. Imaging experiments were performed on day 9–14 after plating when neurons began to show spontaneous Ca^2+^ oscillations.

### Fluorescence calcium measurements

Coverslips containing a monolayer of adherent EA.hy926 or PC12 cells were loaded with the ratiometric Ca^2+^ indicator Fura-2 AM (Molecular Probes) as described previously [28]. Briefly, cells were incubated in standard HBSS solution (NaCl 121 mM; KCl 5.4 mM; MgCl_2_ 0.8 mM; CaCl_2_ 1.8 mM; NaHCO_3_ 6 mM; D-glucose 5.5 mM; HEPES 25 mM, pH 7.3) containing 1 µM Fura-2 ester for 45 min followed by a 30 min de-esterification period in HBSS solution at room temperature. Imaging was performed using a 10× objective with the Discovery-1 by alternately exciting the Fura-2 dye at 340 nm and 380 nm. Emitted light was filtered at 540 nm and collected with a cooled CCD camera (CoolSnap HQ, Roper Scientific). The intracellular Ca^2+^ concentration was calculated from background corrected fluorescence images (F) according to the equation, Ca^2+^ concentration = K_d_×[(R−R_min_)/(R_max_−R)] [29]. The fluorescence at minimum Ca^2+^ (R_min_) was determined by incubating cells with 1 µM ionomycin solution in Ca^2+^ -free buffer containing 1 mM EGTA. The fluorescence at maximum Ca^2+^ (R_max_) was determined by incubating cells with 1 µM ionomycin in Ca^2+^-containing buffer.

In a similar manner, coverslips containing primary hippocampal neurons were loaded with the ratiometric dye Fura-PE3 AM (Calbiochem). Fura-PE3 is a leakage-resistant Ca^2+^ indicator. Cells were incubated in standard HBSS solution containing 2 µM Fura-PE3 AM for 45 min followed by a 30 min de-esterification period in HBSS solution. Imaging was performed at 37°C using the Discovery-1 system by alternately cells loaded with Fura-PE3 at 340 nm and 380 nm. Emitted light >540 nm and collected with a cooled CCD camera and analyzed in the same manner as mentioned for other cell types.

### Imaging Protocols

**Figure 3:**
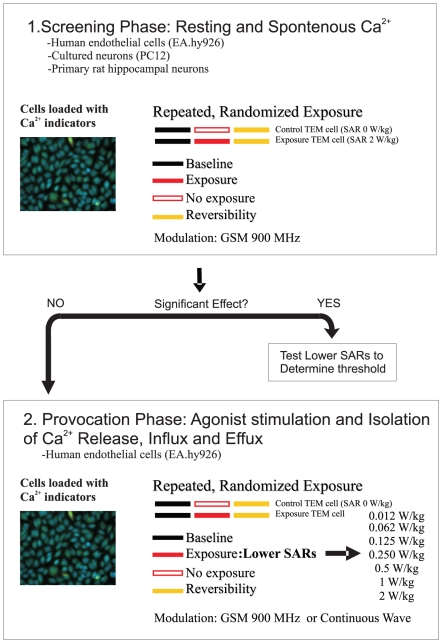
Experimental Flow Chart for Screening RF-Induced Ca^2+^ Effects. The experimental work plan for Ca^2+^ experiments consisted of a Screening Phase (1) and Provocation Phase (2). A screening phase was used to expose all cell types tested to a 2 W/kg 900 MHz GSM field for 30 minutes. If significant changes in Ca^2+^ were observed in the exposure period, additional experiments would be performed at lower SARs to determine the threshold for the effect. If, however, no significant effects were observed in the screening phase, provocation experiments would be undertaken where the Ca^2+^ homeostasis would be deliberately perturbed. For these experiments, the human endothelial cell line (EA.hy926), was chosen and tested at a number of lower SARs, using both continuous wave and GSM modulation conditions.

Imaging experiments were typically carried out in two stages. The experimental design consisted of a screening phase and provocation phase, as illustrated in figure 3. This approach was used due to the large parameter space associated with multiple cell types, SARs and potential measurements to consider.

#### i. Screening Phase

In this set of experiments, imaging protocols consisted of 3 periods lasting 30 minutes each (i.e. 90-minute imaging period in total). A 340 nm and 380 nm image was acquired every 30 seconds during the course of the experiment. Images acquired in the first 30 minutes were used to establish a baseline Ca^2+^ concentration. During the next 30 minutes of image acquisition, cells were exposed to a 900 MHz GSM-modulated RF field, or received no field in a sham exposure condition. A final 30-minutes of imaging was acquired after the exposure period to determine the persistence and reversibility of any putative effects observed in the preceding period. In the case of cultured PC12 and primary hippocampal cells, the vitality and responsiveness of neurons was assessed at the end of the imaging protocol by application of 54 mM KCl. KCl-mediated depolarization causes large, rapid and reversible Ca^2+^ transients in vital neurons. Only cells displaying a robust, reversible response to KCl were included in the analysis presented herein.

#### ii. Provocation Phase

Following the screening phase, a second series of ‘provocation’ experiments was performed. In this case, Ca^2+^ signals were actively induced with a number of pharmacological manipulations to study the effect of RF exposure on various components of cellular Ca^2+^ handling. EA.hy926 cells were chosen for these experiments and imaged every 30 seconds in Ca^2+^-free HBSS solution for 30 minutes to determine baseline Ca^2+^ concentration. The cells were then exposed to a GSM-modulated RF field, or to no field in the sham exposure condition, for 30 minutes.

Ca^2+^ release from intracellular stores was evoked by application of histamine. Whilst passive Ca^2+^ store depletion and Ca^2+^ entry were stimulated by incubation with thapsigargin.

### Statistical Analyses

Statistical analyses were performed using analysis of variance tests (ANOVA) with the SAR of RF exposure and modulation conditions (GSM or CW) as the independent variables. Cytosolic Ca^2+^ concentration was the dependent variable. When cells were compared across different time periods (ie. baseline vs. exposure vs. washout), a mixed model ANOVA with repeated measures was used to analyze significant differences across the time periods and compare this between the exposed and control samples. Dependent variables were distribution tested for normality, homogeneity of variance and sphericity; the latter in the case of repeated measures. In cases where dependent measures violated these assumptions, the data was transformed or non-parametric statistical tools were used. Statistical significance was accepted where p<0.05. All statistical analyses were performed with SPSS 16 for Windows. >150 cells were analysed on each of the coverslips tested.

## Results

### Human Endothelial Cells (EA.hy926)

**Figure 4:**
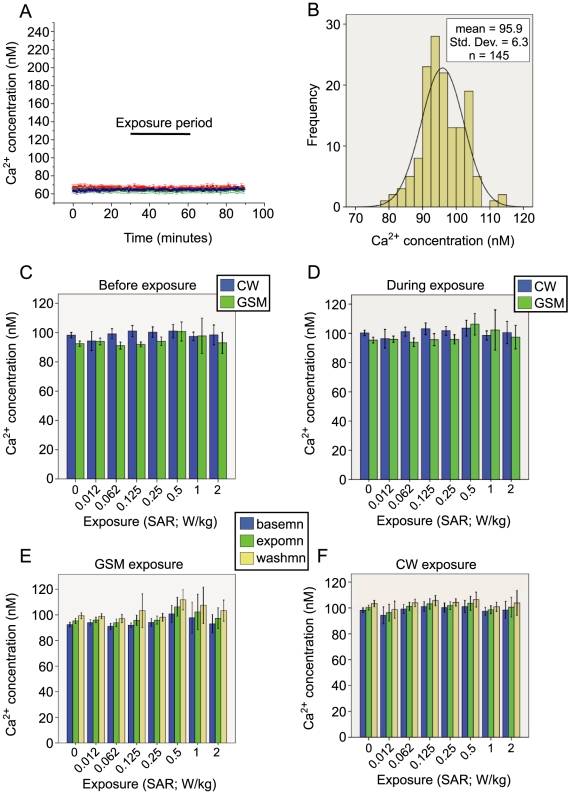
Resting Ca^2+^ Concentration in Human Endothelial Cells Exposed to RF Fields. **A**; Sample traces showing the experimental protocol. Cytosolic Ca^2+^ was measured in EA.hy926 cells exposed to 900 MHz RF at 7 levels of SAR using either GSM-modulated or continuous wave (CW) power. **B**; Cytosolic Ca^2+^ was normally distributed, permitting the use of parametric statistical tools (Kolmogorov-Smirnov Z = 0.739, p = .65, tested against a normal distribution). **C**; No significant differences were noted in the baseline concentration of Ca^2+^ across groups before the exposure period (F(7,145) = 1.15, p = 0.34). **D**; During the exposure period, no significant differences were observed in the mean Ca^2+^ concentration of cells exposed to any of the 7 levels of SAR for a 30-minute exposure period, when compared to unexposed cells (F(7,75) = 2.19, p = .05) (an unexposed sample (SAR of 0 W/kg) was run simultaneously with each exposed coverslide). No interaction effect for modulation was observed, as there was no difference in the mean Ca^2+^ concentration at any SAR in either the GSM or CW exposure conditions (F(7,145) = 1.33, p = 0.24). **E**; Repeated measures analysis of variance was performed using 10-minute means before exposure (basemn), the first ten minutes of exposure (exposmn) and in the final ten minutes after exposure had ceased (washmn), showing that no significant differences existed in Ca^2+^ concentration between the SARs tested for either the GSM (F(7.5,71.78) = 1.77, p = 0.10) or the, **F**, CW power condition (F(7.37,65.32) = 0.21, p = .99). Repeated measures of Ca^2+^ concentration failed Mauchly's test, therefore Greenhouse-Geiser corrected statistics were used.

Cytosolic Ca^2+^ was measured in human endothelial cells exposed to RF fields at different levels of SAR. Coverslides of cells were exposed to 1 of 7 SARs (0.012, 0.062, 0.125, 0.25, 0.5, 1, or 2 W/kg) for durations of 30 minutes with either CW or GSM-modulated RF power (Figure 4A). A control coverslide (SAR = 0) was run in the non-energized TEM cell simultaneously with each exposed sample. The influence of the RF exposure on resting Ca^2+^ was analysed using a mixed model 2×8 factorial ANOVA with three levels of repeated measures, corresponding to three recording periods. The resting intracellular Ca^2+^ concentration of EA.hy926 cells was normally distributed around a mean of 94.51 nM (±6.34) (Figure 4B) (Kolmogorov-Smirnov Z = 0.739, p = .65, tested against a normal distribution). A total of 145 coverslides were analyzed in total with approximately 170 cells per coverslide, for a total of 25,000 cells screened. The cell density of each coverslide was kept constant across the exposure conditions and it was verified that there were no significant differences in culture confluence between exposure conditions *post hoc* (F_(7,144)_ = 0.75, p = 0.63).

There was no significant difference in the baseline Ca^2+^ concentration of coverslides exposed to either the GSM or CW exposure conditions when compared to control coverslides (F_(7,145)_ = 1.15, p = 0.34)(Figure 4C). Furthermore, no significant difference was observed in the Ca^2+^ concentration of coverslides exposed to any of the SARs between 0 and 2 W/kg during the 30-minute exposure period, (F_(7,75)_ = 2.19, p = .05) (Figure 4D). In addition, no significant interaction effect existed between the type of modulation used and the levels of SAR, as there was no significant difference in the mean Ca^2+^ concentration at any SAR in either the GSM or CW exposure conditions (F_(7,145)_ = 1.33, p = 0.24)(Figure 4D).

A further statistical analysis was performed to take advantage of the repeated measurements of Ca^2+^ concentration in the human endothelial cells (Figures 4E and F). A repeated measures analysis of variance was performed using the mean Ca^2+^ level in the 10 minutes before exposure (basemn), the first 10 minutes of exposure (exposmn) and in the final ten minutes after exposure had ceased (washmn). The repeated measures of Ca^2+^ concentration did not meet the assumption of sphericity, having failed Mauchly's test, therefore, Greenhouse-Geiser corrected statistics were used. The mixed model repeated measures analysis of Ca^2+^ concentration showed no significant differences between the unexposed coverslides and any of the SARs, even considering the three time periods, for either the GSM (F_(7.5,71.78)_ = 1.77, p = 0.10), or CW power condition (F_(7.37,65.32)_ = 0.21, p = .99) (Figures 4E and F). To take account of the whole period, rather than the just the 10 minute means, a slope was calculated for the Ca^2+^ concentration for each of the three 30 minute periods (baseline, exposure and washout) and analyzed with the mixed model repeated measures approach. The slopes of the Ca^2+^ concentration in the 30-minute periods were normally distributed (Kolmogorov-Smirnov Z = 1.01, p = 0.26). Analysis of the slopes showed no significant differences between the unexposed coverslides and those exposed to any of the SARs tested, considering the three repeated measures of slope for each sample (F_(9.51, 175.29)_ = 1.64, p = 0.10; results not shown). These results indicate that cytosolic Ca^2+^ concentration in EA.hy926 human endothelial cells neither increases nor decreases in response to a 30-minute exposure to 900 MHz GSM or CW RF power at either 2 W/kg or the six lower SARs tested.

### Agonist-Stimulated Ca^2+^ Signals in Human Endothelial Cells

**Figure 5:**
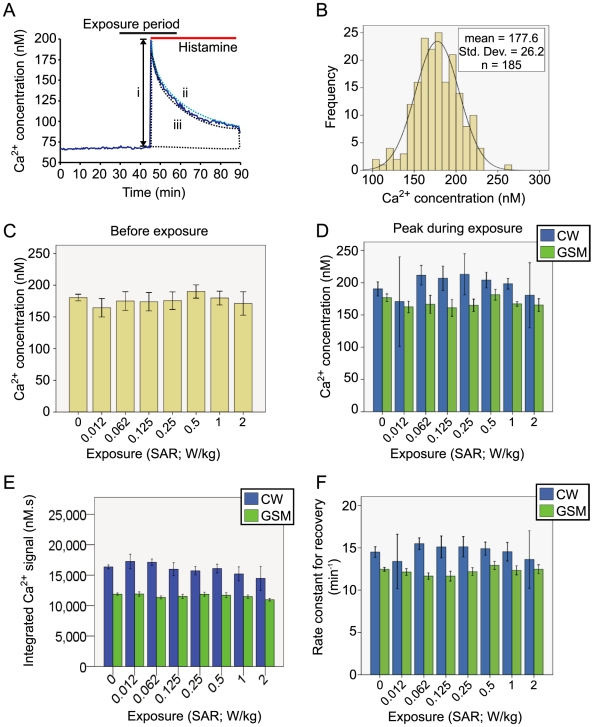
Effect of RF exposure on Histamine-Induced Ca^2+^ Release. **A**; Sample trace showing the experimental protocol. Cytosolic Ca^2+^-release induced by histamine was measured in EA.hy926 cells exposed to 900 MHz RF fields at 7 levels of SAR using either GSM-modulated or continuous wave (CW) power. The peak Ca^2+^-release (**i**), time constant for recovery of the Ca^2+^ signal (**ii**) and integrated Ca^2+^ response (area under the Ca^2+^ curve) (**iii**) was quantified for each cell imaged. **B**; cytosolic Ca^2+^-release was normally distributed, permitting the use of parametric statistical tools (Kolmogorov-Smirnov Z = 0.81, p = .54, tested against a normal distribution). **C**; main effect. When GSM and CW exposures were pooled, no significant difference was observed in the peak Ca^2+^ release of cells exposed to either of the 7 SARs during the 30-minute exposure period, (F(7,184) = 1.10, p = .37). **D**; considered individually, there were no significant differences in Ca^2+^ release in either the GSM (F(7,135) = 1.78, p = .10) or CW conditions (F(7,50) = 1.06, p = 0.41). **E**; No significant differences were observed in the integrated Ca^2+^ signal between control and exposed coverslides at any of the SARs tested in either CW or GSM modulation. (all F<1, p>.10). **F**; no significant differences were observed in the recovery of the Ca^2+^ signals at any of the SARs in either CW or GSM exposed samples (all F<1, n.s.). Repeated measures failed Mauchly's test, therefore Greenhouse-Geiser corrected statistics were used.

In order to test the effect of RF exposure on provoked Ca^2+^ signals, a threshold concentration (1 µM) of the agonist histamine was applied to cause Ca^2+^ release from intracellular stores during the exposure period. The experimental protocol is illustrated in figure 5A. The EA.hy926 cells were exposed to 1 of 7 SARs (0.012, 0.062, 0.125, 0.25, 0.5, 1, or 2 W/kg) for 30 minutes with either CW or GSM-modulated RF power. A control coverslide (SAR = 0) was run in the non-energized TEM cell simultaneously with each exposed sample.

No significant differences were observed between the baseline Ca^2+^ concentration of exposed and sham-exposed coverslides (F_(7,184)_ = 1.53, p = .16). Histamine treatment caused an expected rapid increase in cytosolic Ca^2+^, which peaked at 177 nM (±26 nM) and was normally distributed (Kolmgorov-Smirnov Z = 0.81, p = .54), as shown in Figure 5B. No significant differences were observed in this peak Ca^2+^ response to histamine in coverslides exposed to any of the 7 SARs tested when compared to controls (F_(7,185)_ = 1.23, p = .29); shown in Figure 5C. The modulation condition (CW or GSM) of the RF exposure did not influence the peak histamine-induced Ca^2+^ signal in exposed and control coverslides at any of the SARs tested (F_(7,169)_ = 0.87, p = 0.53) (Figure 5D). There were no significant differences in the integrated Ca^2+^ signal between control and exposed a coverslides at any of the SARs tested in either CW or GSM modulation. (all F<1, p>.10). Finally, no significant differences were observed in the time constant for recovery of the Ca^2+^ signals at any of the SARs in either CW or GSM exposed samples (all F<1, p>.10.) (Figure 5F).

### Ca^2+^ Stores, Efflux and Entry in Human Endothelial Cells

**Figure 6:**
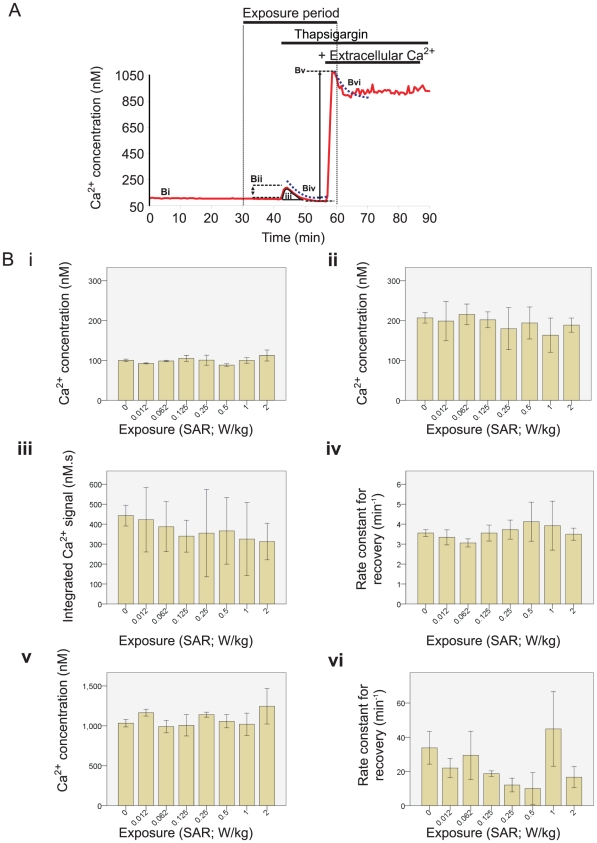
Effect of RF exposure on thapsigargin-induced Ca^2+^Signals. Cytosolic Ca^2+^ was manipulated in EA.hy926 cells through the application of thapsigargin during the exposure period. Cells were exposed to 900 MHz RF fields at 7 levels of SAR with GSM modulated power. **A**; illustration of the protocol used for thapsigargin experiments, showing the various Ca^2+^ signals elicited by thapsigargin and addition of extracellular Ca^2+^. **B**; Analysis of the Ca^2+^ signals evoked by addition of thapsigargin and extracellular Ca^2+^. The numbered panels in **B** refer to the parameters marked on the trace in **Bi**, No significant difference existed in the resting Ca^2+^ prior to RF exposure period. **Bii**, RF exposure at a wide range of SARs showed no influence on the thapsigargin-induced peak Ca^2+^ signal, **Biii**, integrated Ca^2+^ release signal, **Biv**, rate constant for recovery of the Ca^2+^ signal, **Bv**, peak Ca^2+^ entry caused by the addition of extracellular Ca^2+^, or, **Bvi**, the rate constant for recovery of the Ca^2+^ entry signal (all Fs<1, n.s.).

The influence of RF exposure on passive Ca^2+^ efflux from intracellular stores and Ca^2+^ entry was studied using thapsigargin, which disrupts the sequestration of Ca^2+^ by the ER Ca^2+^ATPases. Inhibition of the Ca^2+^ATPases by thapsigargin causes Ca^2+^ to slowly leak from ER stores, eventually leading to their complete emptying. The protocol that we [27] and many others have used for examining thapsigargin-evoked Ca^2+^ signals is depicted in figure 6A. In the absence of extracellular Ca^2+^, thapsigargin causes a transient Ca^2+^ signal. This is due to loss of the finite Ca^2+^ content within the ER and the progressive removal of the ion by plasma membrane ATPases that are insensitive to thapsigargin. The depletion of the ER Ca^2+^ stores activates a Ca^2+^ influx pathway known as ‘store operated Ca^2+^ entry’. As a consequence, the re-addition of Ca^2+^ to the extracellular buffer is associated with a rapid Ca^2+^ entry signal (Figure 6A). All measures obtained in this experimental series were normally distributed, except the rate constant for recovery of the Ca^2+^ entry signal and integrated Ca^2+^ signal.

Cells exposed to GSM RF fields at 1 of 7 SARs (0.012, 0.062, 0.125, 0.25, 0.5, 1 or 2 W/kg) for 30 minutes had similar peak thapsigargin-evoked Ca^2+^ signals (F_(7,57)_ = 0.297, p = 0.95) and amount of total Ca^2+^ sequestered and slowly released from ER stores (F_(7,57)_ = 0.258, p = 0.97) (Fig. 6B). The recovery of thapsigargin-evoked Ca^2+^ signals (largely due to plasma membrane Ca^2+^ATPases) was quantified by fitting mono-exponential decay curves. There were no significant differences in the time constants for the recovery of thapsigargin-evoked Ca^2+^ signals when the RF exposed coverslides were compared to the control samples at any of the SARs tested (F_(7, 57)_ = 0.471, p = .911; Figure 6Biv).

Addition of extracellular Ca^2+^ to thapsigargin-treated coverslides induced a large store-operated Ca^2+^ entry signal, which was not significantly different across the various RF exposure SARs (F_(7,57)_ = 0.568, p = .778; Figure 6Bv). The recovery rate constant of the Ca^2+^ entry signal in exposed coverslides was also not significantly different than control samples (Kruskal-Wallis test χ^2^ = 3.375, p = .19; Figure 6Bvi).

### Cultured Rat Neurons (PC12)

**Figure 7:**
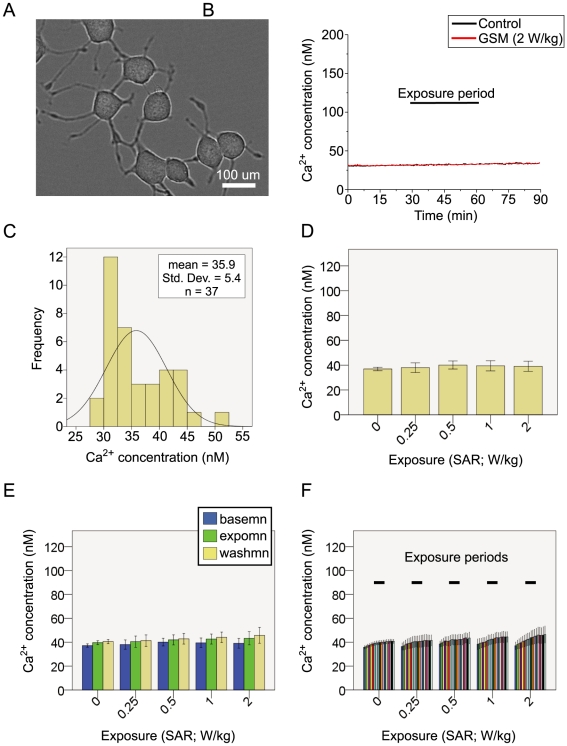
RF Exposure has no influence on Ca^2+^ in PC12 cells. **A**; Image of differentiated cultured rat PC12 cells shown under phase contrast microscopy. **B**; Sample trace showing the experimental protocol. The traces show the Ca^2+^ concentration for two coverslides that were simultaneously imaged. One coverslide was exposed to a GSM RF field of 2 W/kg and other was placed in the non-energized TEM cell. **C**; the Ca^2+^ concentration in PC12 cells was normally distributed. **D**; the mean Ca^2+^ concentration during the exposure period was compared across groups according to SAR. Exposure had no influence on the Ca^2+^ concentration, as there were no differences observed between any of the SARs and the control coverslides (F(4,35) = 0.243, p = 0.912). **E**; the baseline, exposure and washout periods were considered in a repeated measures analysis of variance for changes in Ca^2+^ concentration over these times. No significant differences were found in the change of Ca^2+^ concentration between these periods that were attributable to the SAR of the RF exposure (F(4.5,35.2) = 0.578, p = 0.7, Greenhouse-Geiser corrected). **F**; smaller temporal bins were computed to elucidate any transient effects that might be smeared by averaging over the 30-minute periods. No significant differences were observed in the change of Ca^2+^ concentration over time for the SARs considered, when 5 minute temporal bins were used (F(10.79,83.58) = 0.817, p = .621, Greenhouse-Geiser corrected).

The influence of GSM RF exposure on neuronal Ca^2+^ was first tested with cultured PC12 cells, (Figure 7A). Using a similar exposure paradigm as previously described, cells were imaged to establish a baseline Ca^2+^ concentration and then exposed to one of a range of SARs (0.25, 0.5, 1.0 or 2.0 W/kg). A control coverslide was run in parallel with each experiment in the unenergized TEM cell (SAR = 0). A final 30-minute period was recorded at the end of the exposure to study the persistence of any observed effects. Figure 7B shows a typical experimental trace of the cytosolic Ca^2+^ in a coverslide of PC12 cells exposed to the GSM RF condition with an SAR of 2 W/kg for 30 min, and its matched control sample.

The resting Ca^2+^ concentration of PC12 cells was normally distributed around a mean of 35.9 nM with a SD of 5.43 (Figure 7C). There was no significant difference in the cell density on cover slides across experimental conditions (F_(4,36)_ = 0.05, p = 0.99), nor were there any differences across groups with respect to their resting Ca^2+^ concentration at the start of the experiment (F_(4,36)_ = 0.125, p = 0.972).

Analysis of the mean Ca^2+^ concentration during the exposure period failed to show any influence of RF exposure at the SARs tested when compared to controls (F_(4,35)_ = 0.243, p = 0.912) (Figure 7D). In order to compare the evolution of any exposure effects over time, a mixed model repeated measures ANOVA was performed. Three repeated measures were computed for each sample by binning the Ca^2+^ concentration over the first 5 min of the experiment (basemn), last 5 min of the exposure period (expomn) and final 5 min of the experiment (washmn). No significant effect for RF exposure was observed (F_(4,31)_ = 0.271, p = 0.894), showing that there were no differences in Ca^2+^ concentration across the SARs tested. Interactions between SAR and the repeated measure factor were not observed (F_(4.5,35.2)_ = 0.578, p = 0.7, Greenhouse-Geiser corrected), indicating that neither SAR had an effect on Ca^2+^ concentration at either of the 3 times chosen (Figure 7E). A more complete repeated measure model was also considered, which could discern a transient change in Ca^2+^ concentration as a result of the exposure. The Ca^2+^ concentration was binned into 18 5-minute blocks, allowing another 6 levels of repeated measured per epoch to be considered in the model. No higher order interactions were observed (epoch*block*SAR: F_(10.79,83.58)_ = 0.817, p = .621, Greenhouse-Geiser corrected) with the additional repeated temporal measures of Ca^2+^ concentration added to the model, suggesting that exposure failed to induce any significant changes in Ca^2+^ in PC12 cells at the SARs tested (Figure 7F).

### Cultured Rat Hippocampal Neurons

**Figure 8:**
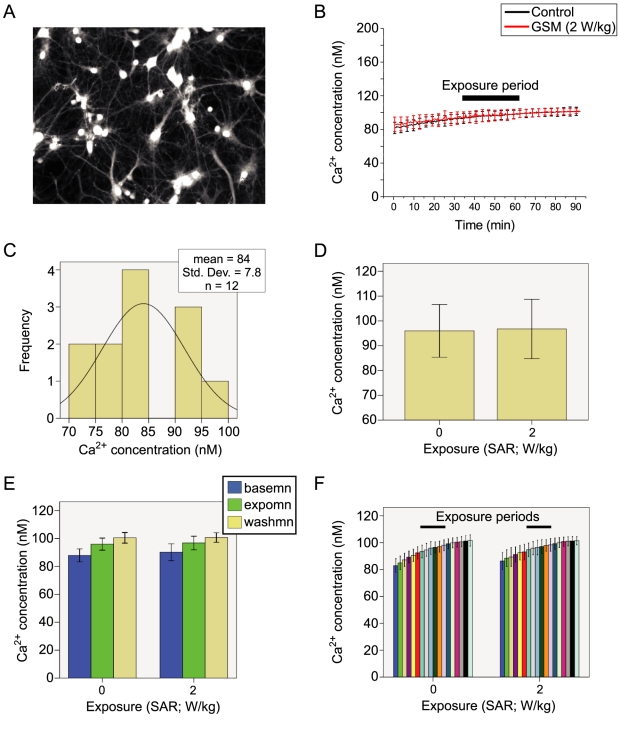
RF Exposure has no influence on Ca^2+^ in Hippocampal neurons. **A**; image of cultured hippocampal neurons loaded with the Ca^2+^ indicator Fura2-PE3. **B**; Sample trace showing the experimental protocol. The recordings from the hippocampal neurons over the experimental time course comprised baseline, exposure and washout periods, each lasting 30 minutes. Neurons were imaged while placed in one of two TEM cells, receiving either a 900 MHz GSM field at an SAR of 2 W/kg or a sham exposure by being in the un-energized TEM cell. **C**; the baseline Ca^2+^ concentration of hippocampal neurons was normally distributed around a mean of 84 nM with a SD of 7.8. **D**; the mean Ca^2+^ concentration of hippocampal neurons for the RF exposure period. No significant difference was observed (F(1,11) = 0.06, p = 0.81) between the GSM 2 W/kg exposed and control samples. **E**; a mixed model repeated measures analysis of variance was performed comparing the baseline, exposure and washout periods between the GSM-exposed and control samples A significant increase in Ca^2+^ concentration was observed across the three time periods (F(1.27,12.68) = 74.93, p<0.01), however, there was no significant interaction with the exposure SAR, indicating that the linear increase over time occurred equally in the hippocampal neurons exposed to the GSM and control conditions (F(1.27,12.66) = 0.65, p = 0.47). **F**; no significant transient changes in Ca^2+^ concentration were observed when the GSM and control conditions were compared with a repeated measures model with 5-minute temporal bins (F(2.11, 21.14) = 0.87, p = 0.44). Greenhouse-Geiser corrected statistics were used.

To screen a class of primary cells from the central nervous system, acutely isolated hippocampal neurons were exposed to GSM RF fields. Dissociated hippocampal neurons form extensive networks in culture, as shown in Figure 8A, which is a fluorescence image of cells loaded with the Ca^2+^ indicator Fura2-PE3. Cultures of hippocampal neurons were placed in the dual TEM cell imaging system and the Ca^2+^ concentration was measured over the course of a 30-minute baseline, 30-minute exposure and 30-minute washout period. At the onset of the exposure period, one coverslide of hippocampal neurons was exposed to a 900 MHz GSM field at an SAR of 2 W/kg, while the other coverslide of neurons served as a matched control sample. A typical experimental trace of the cytosolic Ca^2+^ in a coverslide of hippocampal neurons exposed to the GSM RF condition with an SAR of 2W/kg for 30 min, and its matched control sample is depicted in Figure 8B.

The mean baseline Ca^2+^ concentration for the hippocampal neurons exposed to the control or GSM exposed conditions was normally distributed around a mean of 84 nM with a SD of 7.8 (Figure 8C), permitting the use of parametric statistical tests (Kolmogorov-Smirnov Z = 0.37, p = 0.99).

The mean Ca^2+^ concentration during the exposure period (30–60 min) was compared between hippocampal neurons in the control condition and those receiving the GSM pulse at an SAR of 2 W/kg. No significant difference in Ca^2+^ concentration was observed between the two groups during this period (F_(1,11)_ = 0.06, p = 0.81), as shown in figure 8D. In order to consider any potential changes in the Ca^2+^ concentration between the baseline, exposure and washout periods that might be attributable to the GSM exposure, a mixed model repeated measures analysis of variance was performed. A significant linear increase in Ca^2+^ concentration occurred between the three temporal epochs periods tested (F_(1.27,12.68)_ = 74.93, p<0.01) (Figures 8E and F). However, this occurred equally between the hippocampal neurons exposed to the GSM 2 W/kg field and the control condition, as evidenced by the lack of interaction with the SAR factor (F_(1.27,12.66)_ = 0.65, p = 0.47). Further analyses with repeated measures using smaller temporal increments (5 min) failed to show any transient differences in Ca^2+^ concentration between hippocampal neurons exposed to the GSM 2 W/kg or control condition (F_(2.11, 21.14)_ = 0.87, p = 0.44).

## Discussion

Over the past decades, a substantial number of reports have claimed that electromagnetic field exposure can modify cellular Ca^2+^ binding and homeostasis [18], [30], [31], [32]. The mechanism by which such effects could occur is unknown, but commonly-evoked theories include changes in membrane permeability and free radical generation. With respect to the former concept, it has been suggested that electromagnetic fields can promote Ca^2+^ signals by changing the phase, or packing, of membrane lipids, or by somehow directly modulating Ca^2+^ influx channels [33], [34]. Alternatively, it has been proposed that electromagnetic fields can alter the production or life time of deleterious radical species within cells [35]. Hydroxyl radicals (OH*), for example, are potentially highly toxic moieties that can induce deleterious effects including DNA strand breaks and lipid peroxidation. Exposure of cells to hazardous chemicals or non-ionising radiation can induce the production of such radical species. Links between Ca^2+^ signalling and radicals have been demonstrated in various biological systems, with positive feedback evident in many situations [36]. Interestingly, the spin state of some large organic radicals can be influenced by electromagnetic fields, with a concomitant effect on their reactivity [37], but it is not clear that this applies to the smaller inorganic radicals that impact on Ca^2+^ fluxes.

In contrast to the many reports of demonstrable electromagnetic field effects on Ca^2+^ homeostasis, there are a substantial number of published studies that found no interaction of such radiation with cellular Ca^2+^ signalling systems [38], [39], [40]. These contrasting studies have contributed to a controversial and on-going argument about the ability of different kinds of electromagnetic fields to modulate cellular behaviour. The primary aim of the present study was to use fluorescent Ca^2+^ imaging to establish whether the type of pulsed GSM emissions produced by mobile phone handsets can influence Ca^2+^ signalling under highly-controlled in vitro conditions.

Fluorescent Ca^2+^-binding indicators, such as Fura-2 used in this study, have been extensively employed to monitor cellular Ca^2+^ signals. These indicators can detect rapid changes in Ca^2+^ concentration without significantly altering cellular buffering. In the wider studies of Ca^2+^ signaling in our laboratory, we are routinely able to use such indicators to detect modest Ca^2+^ rises of ∼10 nM amplitude, which represents a 0.1-fold change in Ca^2+^ concentration over baseline and is typically >2 times the standard deviation of the basal signal. In the present study, the use of a sensitive fluorescence imaging system to monitor thousands of cells afforded us the ability to discriminate subtle Ca^2+^ changes.

EA.hy926 human endothelial cells were used to explore specific aspects of Ca^2+^ signalling that might be influenced by the GSM exposure. This cell line was chosen due to several recent reports of their sensitivity to non-thermal GSM RF exposure and speculation that these effects might be mediated by Ca^2+^ signalling events [41], [42], [43]. Evidence from these studies suggests that prolonged exposure (>1 hour) to GSM RF fields causes an up-regulation of calcium-binding proteins and Ca^2+^-associated cytoskeletal and scaffolding proteins. In the present study, we used an acute GSM RF exposure (30 minutes) to investigate modulation of Ca^2+^ homeostasis that might be up-stream of phenotypic changes.

In our experiments, exposure of EA.hy926 cells to GSM RF fields across a wide range of SARs did not lead to any significant changes in the homeostasis of cytosolic Ca^2+^. This was the case when the RF field was presented in the form of pulsed 900 MHz GSM signals similar to those emitted from mobile handsets or when presented as un-modulated 900 MHz RF power. In addition, RF exposure had no influence on the kinetics, amplitude, clearance or re-sequestration of Ca^2+^ released from intracellular stores caused by the application of pharmacologic agonists. Changes in Ca^2+^ homeostasis can therefore be ruled out in any potential biological effects observed in EA.hy926, at least in experiments using a similar duration (30 minutes) and SARs of up to 2 W/kg.

To explore the possibility that different mammalian cell types have varying sensitivity to RF exposure, experiments were repeated using cultured PC12 cells and neurons acutely isolated from the hippocampii of rats. PC12 cells were chosen as they had previously been shown to exhibit transient changes in gene expression in response to GSM RF fields, with modulation of Ca^2+^ proposed as a potential mechanism [44]. Hippocampal neurons were chosen for these studies as they have been shown to exhibit changes in excitability when exposed to RF fields in brain slices [20]. No effects of GSM RF exposure on cytosolic Ca^2+^ were observed for either of these cell types in any of the conditions tested.

We conclude that under the conditions employed in our experiments, and using a highly-sensitive assay, we could not detect any consequence of RF exposure. How then can one explain the published results showing that RF fields influence cellular Ca^2+^ signalling? The majority of these results can be traced to one or two groups who used a very specific biological preparation; whole or excised chick brain. There has been much criticism of this work. In particular, in the physiological state of the cells located deep within tissues, which would have poor oxygen perfusion, energy metabolism and homeostasis. It is plausible that the compromised physiology of such tissue samples is critical for observation of RF-induced effects on Ca^2+^. If this were the case, it would explain why improvements in the methodology and tissue conditions led to failed replications of this work [12].

An important point in considering the original experiments by Adey and colleagues is the distinction between the ELF amplitude-modulated RF signals used in their studies and the pulsed GSM RF fields produced by modern mobile phones. GSM pulsed RF signals are digital, whereas the original RF fields that induced changes in Ca^2+^ were analogue, amplitude-modulated fields. In fact, a key finding of Adey's work was that the analogue modulation within specific frequency windows (∼16 Hz; see *Introduction*) was critical to the observation of these effects. In any exposure resulting from current mobile phone technology, there is no 16 Hz modulation in the pulsed GSM signal. There is, however, evidence that handsets can generate EMF in the ELF range due to their battery currents [45], [46]. The ELF-EMF produced by the power use of handsets will likely differ considerably between models, due to their various styles and geometries, and it will therefore be difficult to quantify. The GSM exposure signal used in our experiments did not contain an ELF-EMF component.

Our experiments focused on the potential influence of GSM RF exposure on Ca^2+^ homeostasis and for this reason we studied Ca^2+^ signals near the steady-state or following controlled perturbations. Ca^2+^ oscillations are also observed in some types of cells, but none were observed in any of the cells studied under our experimental conditions. We considered this as an advantage in the present study because it simplified a part of the analysis; however, Ca^2+^ oscillations could be a sensitive measure of RF field effects. Future research might therefore focus on an oscillatory cell type to evaluate whether such oscillations are influenced.

Although acute changes in intracellular Ca^2+^ were not observed in the cells that were exposed to GSM RF in this study, it does not preclude that there may biological responses mediated through other signal transduction pathways. Indeed, recent work has shown that short-term GSM RF exposure (5–30 min) may stimulate MAPK (mitogen-activated protein kinase) cascades [47]. Therefore, perhaps activities such as the RAF/ERK1/2 signalling pathway, and not Ca^2+^ concentration, is the most sensitive measure of GSM RF-EMF effects.
